# Characterization of Metastasis Formation and Virotherapy in the Human C33A Cervical Cancer Model

**DOI:** 10.1371/journal.pone.0098533

**Published:** 2014-06-02

**Authors:** Ulrike Donat, Juliane Rother, Simon Schäfer, Michael Hess, Barbara Härtl, Christina Kober, Johanna Langbein-Laugwitz, Jochen Stritzker, Nanhai G. Chen, Richard J. Aguilar, Stephanie Weibel, Aladar A. Szalay

**Affiliations:** 1 Institute of Biochemistry, University of Wuerzburg, Wuerzburg, Germany; 2 Genelux Corporation, San Diego Science Center, San Diego, California, United States of America; 3 Department of Radiation Medicine and Applied Sciences, Rebecca & John Moores Comprehensive Cancer Center, University of California, San Diego, California, United States of America; 4 Genelux GmbH, Bernried, Germany; 5 Rudolph Virchow Center for Experimental Biomedicine and Institute for Molecular Infection Biology, University of Wuerzburg, Wuerzburg, Germany; Virginia Commonwealth University, United States of America

## Abstract

More than 90% of cancer mortalities are due to cancer that has metastasized. Therefore, it is crucial to intensify research on metastasis formation and therapy. Here, we describe for the first time the metastasizing ability of the human cervical cancer cell line C33A in athymic nude mice after subcutaneous implantation of tumor cells. In this model, we demonstrated a steady progression of lumbar and renal lymph node metastases during tumor development. Besides predominantly occurring lymphatic metastases, we visualized the formation of hematogenous metastases utilizing red fluorescent protein (RFP) expressing C33A-RFP cells. RFP positive cancer cells were found migrating in blood vessels and forming micrometastases in lungs of tumor-bearing mice. Next, we set out to analyze the influence of oncolytic virotherapy in the C33A-RFP model and demonstrated an efficient virus-mediated reduction of tumor size and metastatic burden. These results suggest the C33A-RFP cervical cancer model as a new platform to analyze cancer metastases as well as to test novel treatment options to combat metastases.

## Introduction

Metastatic spread of tumors is a multistage process during which malignant cells disseminate from the primary tumor to distant organs [1]. Tumor cells migrate via two major routes: The lymphatic system and the blood circulation. During lymphatic metastasis, a common feature for most carcinomas, tumor cells leave the original tumor site and migrate after settlement in regional lymph nodes to distant ones [2]. Diagnosis of lymph node metastases is of major importance. Even though, lymph node metastases themselves are rarely life threatening they indicate the state of tumor progression [3]. Most cancer deaths are due to development of metastases. Therefore, a broader understanding of the biology of metastases is necessary. Three major platforms are currently used to develop *in vivo* metastasis models. These include chemical induction, whereby carcinogens are administered to induce tumorigenesis and metastasis, syngeneic and xenograft transplant models that involve the transplantation of tumor cells/tissue into murine hosts and genetically engineered mouse models [4]–[5]. Here, we are working with a xenogeneic spontaneous transplant model of metastasis. A number of xenogeneic metastasis models have been generated during the last years, for example, for prostate [6]–[8], colorectal [9]–[10], breast [11]–[12], gastric [13]–[14] or renal cell [15] carcinomas. Additionally, there are also some models of human papillomavirus (HPV) positive cervical cancer described [16]–[18].

In the course of a cervical cancer screening study (HPV positive and HPV negative cell lines) for oncolytic virotherapy the effects of the oncolytic vaccinia virus GLV-1h68 on these cell lines was analyzed. The results indicate that the presences and the copy number of HPV DNA in cervical cancer cells did not have an impact on the therapeutic efficacy of GLV-1h68 (Table S1). Furthermore we discovered the formation of lymph node metastases after subcutaneous implantation of C33A HPV-negative human cervical cancer cells in immunocompromised mice. Therefore, in this study, we focused on the characterization of the metastasizing ability of this cell line, which has not been described in the literature so far. We analyzed the lymphatic and the hematogenous spread of C33A-RFP cells in nude mice after subcutaneous implantation of tumor cells, whereby lymphatic circulation represented the main route of metastasizing tumor cells. Moreover, a steady progression of lymph node metastases in correlation with the tumor growth was demonstrated.

In addition, we analyzed oncolytic virotherapy of C33A tumors and metastases as a novel treatment option. Oncolytic viruses are able to selectively replicate in cancer cells, resulting in destruction of tumor tissue, but leaving healthy tissues unharmed [19]. Here, we focused on studying the effect of the previously described attenuated, recombinant vaccinia virus GLV-1h68 [20]–[21]. The therapeutic effect of this virus on primary tumors has been shown for many carcinomas such as breast, pancreatic or prostate cancer [21]–[23]. Furthermore, we recently showed a therapeutic potential of GLV-1h68 in treating lymphatic and hematogenous metastases originating from the human prostate carcinoma cell line PC-3 [22], indicating that GLV-1h68 might be able to eradicate metastases in the C33A-model as well. Indeed, here we demonstrated a drastic virus-mediated reduction of C33A-RFP tumors and their metastatic burden.

## Materials and Methods

### Cell lines

The HPV-negative human cervical cancer cell line C33A was cultured in DMEM High Glucose (PAA Laboratories, Cölbe, Germany) supplemented with 10% FCS (PAA Laboratories, Cölbe, Germany) and 1% gentamicin solution (PAA Laboratories, Cölbe, Germany). The C33A cell line was kindly provided by Frank Stubenrauch, PhD (UKT, University of Tübingen; [24]). Authenticity of C33A cells was verified by the DSMZ GmbH (Leibniz Institute DSMZ-German Collection of Microorganisms and Cell Cultures, Braunschweig, Germany). C33A-RFP cells were cultured under same conditions except for adding 5 µg/mL blasticidin. Human epithelial kidney cells (293FT) were obtained from Invitrogen GmbH (Karlsruhe, Germany) and cultured in RPMI 1640 supplemented with 10% FCS and 2 mM L-glutamine (PAA Laboratories, Cölbe, Germany).

### Generation of C33A-RFP cells

The cDNA sequence of the red fluorescent protein (*mRFP1*) was inserted into the C33A cell genome using Vira Power Lentiviral Expression System Kit (Invitrogen GmbH, Germany) in accordance with the manufacturer's instructions. The *mRFP1*-encoding plasmid pCR-TK-SEL-mRFP was provided by Q. Zhang (Genelux Corporation, San Diego) and used to generate the *mRFP1-*containing lentiviral vectors as described previously [25]. Replication-incompetent *mRFP1*-encoding lentiviruses were produced in 293FT cells by co-transfection of the plasmids pLP1, pLP2, pLP/VSVG that supply lentiviral structural and replication proteins and the pLENTI6/V5-DEST-mRFP expression plasmid using Lipofectamine™2000. After transduction of C33A cells with mRFP-encoding lentiviruses and blasticidin (5 µg/mL) selection, one stable RFP expressing C33A clone was selected.

### Virus strain

The attenuated vaccinia virus strain GLV-1h68 was previously described by Zhang *et al*. [21]. Three expression cassettes encoding for *Renilla* luciferase-GFP fusion protein, β-galactosidase, or β-glucuronidase were recombined into the *F14.5L*, *J2R* and *A56R* loci, respectively, of the parental LIVP virus genome.

### Ethics statement

All animals were cared for and handled in strict accordance with good animal practices as defined by the national and local animal welfare bodies (Guide for the Care and Use of Laboratory Animals published by the National Institutes of Health and the German Animal Welfare Act “TierSchG”). Experimental protocols were approved by the government of Unterfranken, Germany (protocol numbers 55.2-2531.01-17/08 and 55.2-2531.01-25/12) and/or the Institutional Animal Care and Use Committee (IACUC) of Explora BioLabs, located in San Diego Science Center (San Diego, USA) (protocol numbers: EB08-003; EB11-025).

### Tumor implantation and virus administration

Tumors were generated by implanting 5×10^6^ C33A-RFP cells in 100 µL PBS subcutaneously into the right abdominal flank of 6-8 weeks old female athymic nude *Foxn1^nu^* mice (Harlan Winkelmann GmbH, Borchen, Germany). Tumor and lymph node volume was monitored in two dimensions using a digital caliper and calculated as [(length)×(width)^2^×0.52]. Tumor volume was measured in living mice, whereas lymph node volume was measured *post mortem*, after opening the abdomen. A lymph node was defined to be enlarged when the maximal diameter exceeded 3 mm. For studying the effect of oncolytic virotherapy a single dose of 5×10^6^ plaque forming units (pfu) of GLV-1h68 in 100 µL PBS was injected intravenously (i.v.) into C33A-RFP tumor-bearing mice, after the tumor volume reached 200–250 mm^3^. Mice were sacrificed according to the Guidelines for Euthanasia of Rodents using carbon dioxide.

### Fluorescence imaging

Images of C33A-RFP tumor-bearing mice were taken with the Maestro EX Imaging System (Caliper, Hopkinton, MA, USA). Fluorescence imaging of tumors, kidneys, lungs and lymph nodes of C33A-RFP tumor-bearing mice was performed with a MZ16 FA Stereo-Fluorescence Microscope (Leica, Wetzlar, Germany). For imaging of mice with the Maestro EX Imaging System, animals were anesthetized using 2-3% isoflurane. Digital images were processed with Photoshop 7.0 (Adobe Systems, Mountain View, USA).

### Measurement of fluorescence intensity

Measurement of the fluorescence intensity of the RFP signal in lymph nodes and kidneys of C33A-RFP tumor-bearing mice was performed using ImageJ (http://rsbweb.nih.gov/ij). RGB-images of the RFP signal of kidneys and lymph nodes were converted into 8-bit gray scale with an intensity range from 0–255. The fluorescence intensity represents the average brightness of all RFP related pixels.

### Immunohistochemistry

For histology, tumors, lymph nodes and kidneys were excised and fixed for 16 h in 4% paraformaldehyde/PBS, pH 7.4. Preparation of 100 µm sections and labeling procedures were performed as described previously [26] using the Leica VT1000 Vibratom (Leica, Heerbrugg, Switzerland). After labeling, tissue sections were mounted in Mowiol 4–88 (Sigma-Aldrich, Taufkirchen, Germany). For preparation of 10 µm sections tissue samples were sectioned with the cryostat 2800 Frigocut (Leica, Wetzlar, Germany). After dehydration in 10% and 30% sucrose (Carl Roth, Karlsruhe, Germany) specimens were embedded in Tissue-Tek O.C.T. (Sakura Finetek Europe B.V., Alphen aan den Rijn, Netherlands). Cryo-sections were stored at −80°C and incubated with primary antibodies for 1 h. After washing with PBS, sections were stained for 1 h with secondary antibodies and finally mounted in Mowiol 4–88.

### Antibodies and reagents

Endothelial blood vessels were stained with a hamster monoclonal anti-CD31 antibody (Chemicon International, Temecula, USA; MAB1398Z) and endothelial lymph vessels with a rabbit polyclonal anti-LYVE-1 antibody (Abcam, Cambridge, UK; ab14917). Nuclei were Hoechst 33342-labeled (Sigma Aldrich, Taufkirchen, Germany). DyLight488-conjugated secondary antibodies (donkey) were obtained from Jackson ImmunoResearch (Pennsylvania, USA). Primary and secondary antibodies were diluted 1∶100 in PBS/0.3% Triton-X-100.

### Fluorescence microscopy

Images of tumor, lymph node and kidney sections were captured with the following microscopes: A stereo-fluorescence microscope MZ16 FA (Leica) equipped with a digital CCD camera and the Leica IM1000 4.0 software (1300×1030 pixel RGB-color images), a TCS SP2 AOBS confocal laser microscope (Leica) equipped with the LCS 2.16 software (1024×1024 pixel RGB-color images) and a Axiovert 200 M microscope (Zeiss) with Axiovision 4.5 software (1388×1040 pixel gray scale images), respectively. Digital images were processed with Photoshop 7.0 (Adobe Systems, USA) and merged to yield pseudo-colored pictures. Images of cells seeded in 24 well plates were captured either with the stereo-fluorescence microscope MZ16 FA (Leica) or with the Axiovert 200 M microscope (Zeiss).

### Preparation of single cell suspensions and FACS analysis

For preparing single cell suspensions of tumors, lumbar and renal lymph nodes, lungs and kidneys of C33A-RFP tumor-bearing mice tissues were weighed, minced and incubated individually in DMEM High Glucose media supplemented with 2% FCS, 10.000 U/mL Collagenase I (Sigma, Steinheim, Germany) and 5 MU/mL DNase I (Calbiochem, Darmstadt, Germany) at 37°C. Tumor tissues were incubated for 35 min, LN and RN tissues for 30 min, kidneys for 20 min and lungs for 15 min. Subsequently, tissues were passed through 70 µm nylon mesh filters (BD Biosciences, Erembodegem, Belgium) and transferred to DMEM High Glucose media supplemented with 2% FCS. After centrifugation (1000 g, 10 min) pellets were resuspended in 2x volume of PBS/2% FCS, regarding to tissue weight. Subsequent, 100 µL of the single cell suspensions were analyzed using Accuri C6 Cytometer and FACS analysis software CFlow Version 1.0.227.4 (Accuri Cytometers, Inc. Ann Arbor, MI, USA). Cells were gated according to their size (FSC) and granularity (SSC). Furthermore, 100 µL of the single cell suspensions were seeded into 24 well plates in 1×10^−1^ to 1×10^−4^ dilutions. Dilutions were prepared in DMEM High Glucose media supplemented with 10% FCS. Three days after cell suspension seeding media was supplemented with 5 µg/mL blasticidin, to select C33A-RFP cells. Cell washing and media renewal were performed twice a week. After one week of blasticidin selection C33A-RFP cells in the 24 well plates were stained with crystal violet for 3 h, washed and dried. Afterwards, cell colonies were counted.

### Statistical analysis

A two-tailed Student's *t* test was used for statistical analysis. *P* values of ≤0.05 were considered statistically significant. Asterisks indicate a significant difference between experimental groups (* indicates p≤0.05; ** indicates p≤0.01; *** indicates p≤0.001).

## Results

### Growth kinetics of subcutaneous C33A-RFP tumors and lymph node metastases formation

**Figure 1:**
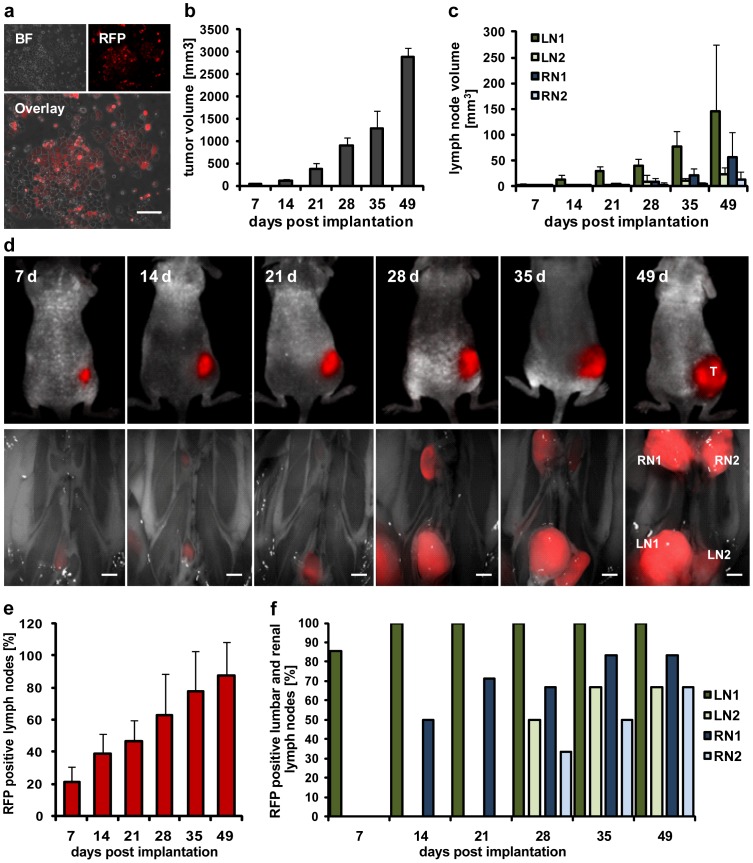
Formation of tumors and lymph node metastases after subcutaneous implantation of 5×10^6^ C33A-RFP cells. Tumors and lymph nodes of six to seven mice per time point were analyzed (7 and 21 dpti: n = 7; 14, 28, 35 and 49 dpti: n = 6). **a** Bright field (BF), RFP and overlay images of C33A-RFP cells, scale bar represents 100 µm. **b** Time curve of C33A-RFP tumor growth. **c** Volume of lumbar and renal lymph nodes over time. **d** Representative images of tumors (upper row) and corresponding lymph nodes (lower row) of C33A-RFP tumor-bearing mice at the indicated days post tumor cell implantation. Images of tumors (T) were taken of living mice using the Maestro EX Imaging System. Imaging of lumbar (LN1, LN2) and renal (RN1, RN2) lymph node metastases in the abdomen of tumor-bearing mice was performed *post mortem*, after opening the abdomen and removing organs. Scale bars represent 2 mm. **e** Percentage of all lymph nodes positive for RFP over time. **f** Percentage of LN1, LN2, RN1 and RN2, respectively, positive for RFP per time point.

An observed enlargement of lumbar and renal lymph nodes in C33A tumor-bearing mice indicated the presence of metastasized tumor cells. To enable visualization of metastatic C33A cell spread, the cDNA sequence of the red fluorescent protein (*mRFP1*) was inserted into the C33A cell genome. Expression of RFP in C33A cells was confirmed by fluorescence microscopy (Fig. 1a). Subsequently, 5×10^6^ C33A-RFP cells were subcutaneously (s.c.) implanted into the right flank of nude mice. Every week up to 49 days post implantation six to seven C33A-RFP tumor-bearing mice were examined. As a part of these analyses volumes of tumors, lumbar (LN1, LN2) and renal (RN1, RN2) lymph nodes were measured (Fig. 1b+c) and fluorescence imaging of tumors and lymph node metastases was performed (Fig. 1d). We showed a steady increase of the tumor volume from week to week after implantation of C33A-RFP cells. At the same time, the volumes of lumbar and renal lymph nodes were increasing as well, indicating a possible tumor cell colonization process resulting in lymph node volume expansion. Fluorescence imaging confirmed RFP signals in the enlarged lymph nodes, demonstrating C33A-RFP metastases. Notably, the volume of LN1, the lumbar lymph node located closest to the primary C33A-RFP tumor, was increasing first and largest, as would be expected for regional lymph node metastases.

Moreover, fluorescence imaging during the time course of 49 days revealed a steady increase of the amount of lymph nodes positive for RFP after tumor cell implantation, starting with 21% RFP positive lymph nodes at day 7. The amount increased to 88% lymph nodes positive for RFP expression at the end of the experiment (Fig. 1e). Furthermore, the percentage of RFP positive LN1, LN2, RN1 and RN2 was analyzed per time point. At day 7 post implantation 86% of all detected LN1s were positive for RFP, whereas no RFP signal was detectable in LN2, RN1 or RN2 leading to the assumption that metastatic colonization with C33A-RFP cells occurs first in the regional lymph node LN1. Fourteen days post tumor cell implantation (dpti) all detected LN1s (100%) and 50% of RN1s were positive for RFP, indicating that after metastasizing to LN1 the C33A-RFP cells spread to the next local lymph node (renal lymph node RN1). Metastasized C33A-RFP cells in LN2 and RN2 were first detected at day 28 post implantation (Fig. 1f). Taken together, we showed a continuous progression of metastasis of lumbar and renal lymph nodes coinciding with the C33A-RFP tumor growth from week to week after s.c. tumor cell implantation.

### C33A-RFP tumor cells metastasize via both the lymphatic and the hematogenous routes

**Figure 2:**
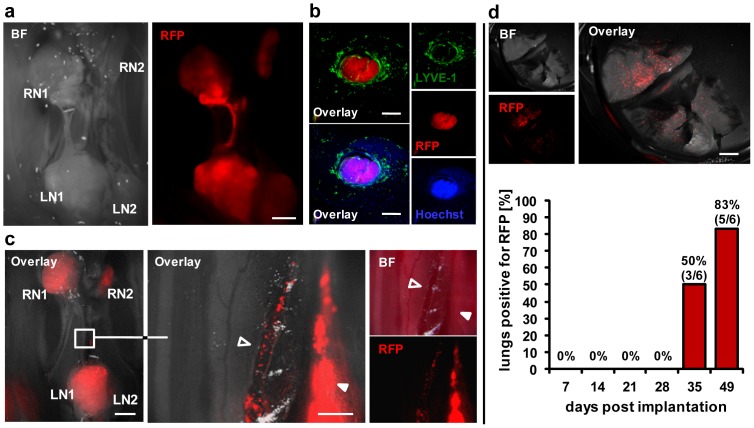
Metastatic migration of the C33A-RFP cells in tumor-bearing nude mice. **a** Migration of C33A-RFP cells in a lymph vessel connecting LN1 and RN1 42 dpti. Scale bars represent 2 mm. **b** LYVE-1 staining of 100 µm cross sections of the part between LN1 and RN1. Scale bars represent 200 µm. **c** Migration of C33A-RFP cells in a lymph (filled arrowhead) and in an erythrocyte containing blood vessel (open arrowhead) 32 dpti. Scale bars represent 2 mm (left) and 500 µm (right). **d** RFP signals in lungs of C33A-RFP tumor-bearing mice. Above: representative image of a lung 42 dpti. Scale bar represents 2 mm. Below: percentage of lungs tested positive for RFP spots over time. Lungs of six to seven mice per time point were examined (7 and 21 dpti: n = 7; 14, 28, 35 and 49 dpti: n = 6).

During our microscopic studies we detected the presence of C33A-RFP cells in lymph vessels connecting lumbar and renal lymph node metastases in C33A-RFP tumor-bearing mice, thereby demonstrating the lymphatics as route of secondary metastasis in this model (Fig. 2a, b).In addition, circulating C33A-RFP cells were also detected in erythrocyte containing blood vessels located next to LN-RN-connecting lymph vessels, reflecting the hematogenous route of metastatic migration of tumor cells (Fig. 2c). Notably, fluorescence imaging revealed RFP signals in lungs of C33A-RFP tumor-bearing mice which usually is the first organ to get metastasized in case of metastatic spread via the hematogenous route. Up to day 28 post tumor cell implantation no lung was found to be positive for RFP, whereas at day 35 three out of six and at day 49 five out of six lungs were tested positive for RFP spots (Fig. 2d).

In summary, we clearly demonstrated that C33A-RFP cells are using the lymphatic as well as the hematogenous routes for metastatic spread in s.c. implanted tumor-bearing nude mice.

**Figure 3:**
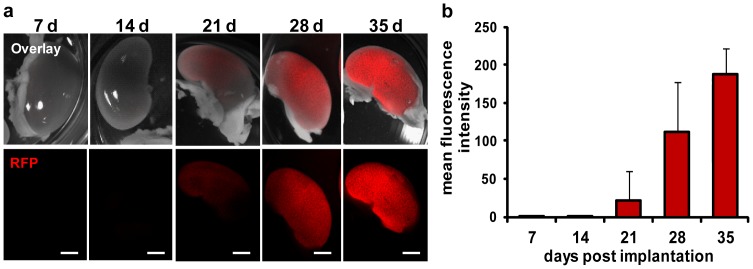
RFP signals in kidneys of C33A-RFP tumor-bearing mice 7, 14, 21, 28 and 35 dpti. Six to seven mice were analyzed per time point (7 and 21 dpti: n = 7; 14, 28 and 35 dpti: n = 6). **a** Representative images of RFP signal in kidneys. Upper row: overlay of bright field and RFP images. Lower row: images of RFP signal. Scale bars represent 2 mm. **b** Mean fluorescence intensity of the RFP signal in kidneys of C33A-RFP tumor-bearing mice over time.

**Figure 4:**
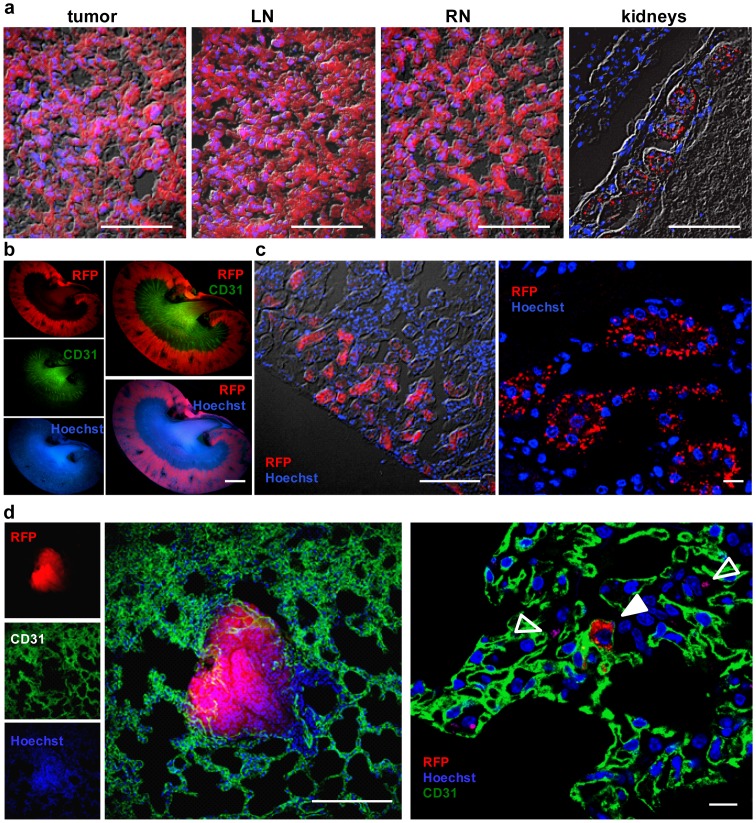
Histological analysis of tumors, LNs, RNs and kidneys of C33A-RFP tumor-bearing mice. Nuclei in a, b and c were stained with Hoechst dye. **a** Overlays of bright field, RFP and Hoechst images of 10 µm sections of a tumor, LN, RN and kidney 31 dpti. Scale bars represent 50 µm. **b** Images of 100 µm sections of a kidney 31 dpti, stained with anti-CD31 antibody and Hoechst dye. Scale bar represents 2 mm. **c** 10 µm kidney section stained with Hoechst dye. Right: confocal image. Scale bars represent 100 µm (left) and 10 µm (right). **d** CD31 and Hoechst staining of 100 µm lung sections 42 dpti. Filled arrowhead: in a blood vessel migrating C33A-RFP cell; empty arrowhead: RFP positive fragments. Scale bars represent 250 µm (left) and 50 µm (right). All images are representative examples.

Furthermore, we observed an accumulation of the RFP signal in kidneys of these mice by fluorescence imaging during the time course of 49 days (Fig. 3). Histological analysis of tumors, LNs and RNs of tumor-bearing mice 31 dpti revealed an organized structure of RFP expressing C33A cells in tumors and lymph node metastases, which was not true for RFP in kidneys (Fig. 4a). Furthermore, we showed that the renal RFP signals were located mainly in the cortex and CD31 labeled blood vessels in the medulla and pelvis of the kidneys (Fig. 4b). Images of the renal cortex with higher magnification revealed RFP accumulation in nephrons, whereby RFP seemed to be located cell-independently in spot-like patterns (Fig. 4c). No micrometastases were observed in kidneys. Therefore, we assume that the strong RFP signal in kidneys of C33A-RFP tumor-bearing mice might be caused by deposition of RFP in renal cortex, after blood filtering in nephrons. In contrast to what we observed in the kidneys, micrometastases were detected in lungs of C33A-RFP tumor-bearing mice 42 dpti (Fig. 4d left). Moreover, single C33A-RFP cells were found in blood vessels of lungs as well as RFP fragments similar to those observed in the renal cortex of the kidneys (Fig. 4d right).

Taken together, histological analyses revealed a clear, organized structure of RFP expressing C33A cells in tumors and lymph node metastases, whereas the RFP signal in kidneys seemed to be mainly the result of protein deposition and not only of metastasized RFP positive tumor cells. RFP signal in lungs seemed to be caused by both, metastasized and settled C33A-RFP cells and RFP deposition.

### Detection of viable C33A-RFP cells in tumors, LNs, RNs, lungs and kidneys of tumor-bearing mice

**Figure 5:**
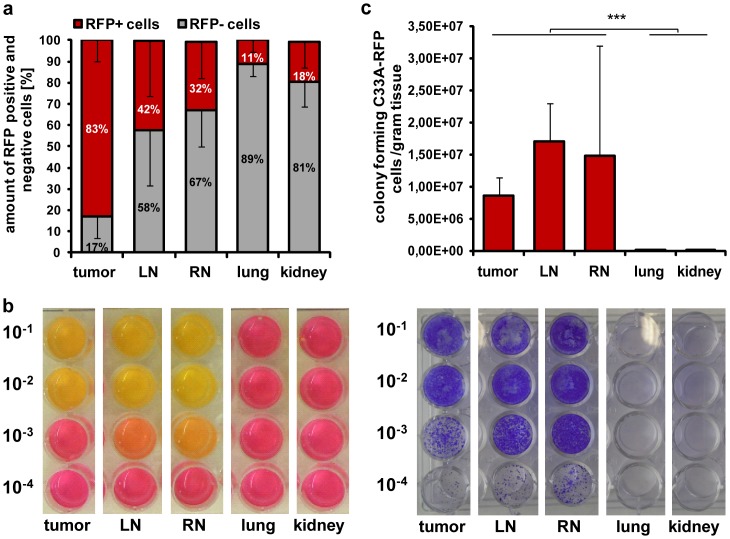
Analysis of tumor, LN, RN, lung and kidney single cell suspensions from C33A-RFP tumor-bearing mice 49 dpti. Tissues of all 5 mice were analyzed (n = 5). **a** Amount of RFP positive (RFP+) and negative (RFP-) cells in single cell suspensions of tumor, LN, RN, lung and kidneys. 10,000 events were analyzed by FACS. **b** Growth of different dilutions (10^−1^–10^−4^) of C33A-RFP single cell suspensions in wells of 24-well plates after one week of blasticidin selection. Left image: media consumption in wells containing 2 days old media. Right image: wells after staining with crystal violet. **c** Numbers of colony forming C33A-RFP cells per gram tumor, LN, RN, lung and kidney, respectively. Cell colonies were counted for each organ after blasticidin-selection and crystal violet staining.

To further investigate whether the RFP signal in tumors, LNs, RNs, lungs and kidneys of C33A-RFP tumor bearing mice resulted from viable C33A cells, single cell suspensions of these tissues of 5 mice 49 dpti were prepared and analyzed by FACS. We found that 83% of the cells in tumor cell suspensions were positive for RFP, 42% in LN, 32% in RN, 11% in lung and 18% in kidney suspensions (Fig. 5a). Hence, RFP positive tumor cells were detectable in all analyzed tissues of C33A-RFP tumor-bearing mice.

To determine how many of these RFP positive cells were indeed viable tumor cells, we performed a blasticidin selection assay. After one week of blasticidin selection media consumption (indicated by changing of the color of phenol red from red to yellow) correlated with the amount of viable cells (Fig. 5b, left). Crystal violet staining showed additionally that tumor, LN and RN suspensions contained more viable C33A-RFP cells than lung and kidney suspensions (Fig. 5b, right). Only a few positive C33A-RFP cell colonies were observed in wells of lung and kidney suspensions in 10^−1^ dilutions, whereas RFP positive cells were noticed in all dilutions of the tumor, LN and RN suspensions.

Subsequently, the stained C33A-RFP cell colonies were counted in the appropriate dilutions and the number of colony forming C33A-RFP cells per gram tissue was determined for tumors, metastases and organs. It turned out that 8.6×10^6^ (±2.9×10^6^) colony forming C33A-RFP cells were detected per gram tumor tissue, 1.7×10^7^ (±5.9×10^6^) in LN and 1.5×10^7^ (±1.7×10^7^) in RN. In contrast to this only 4.8×10^3^ (±9.7×10^3^) colony forming C33A-RFP cells were detected in lungs and 8.1×10^3^ (±1.7×10^4^) in kidneys (Fig. 5c).

Thus, we demonstrated a significant lower amount of viable C33A-RFP cells in lungs and kidneys than in tumors and lymph node metastases of tumor-bearing mice, revealing that C33A-RFP metastases formation occurs mainly via the lymphatic route.

### Combating metastases in the C33A-RFP model

**Figure 6:**
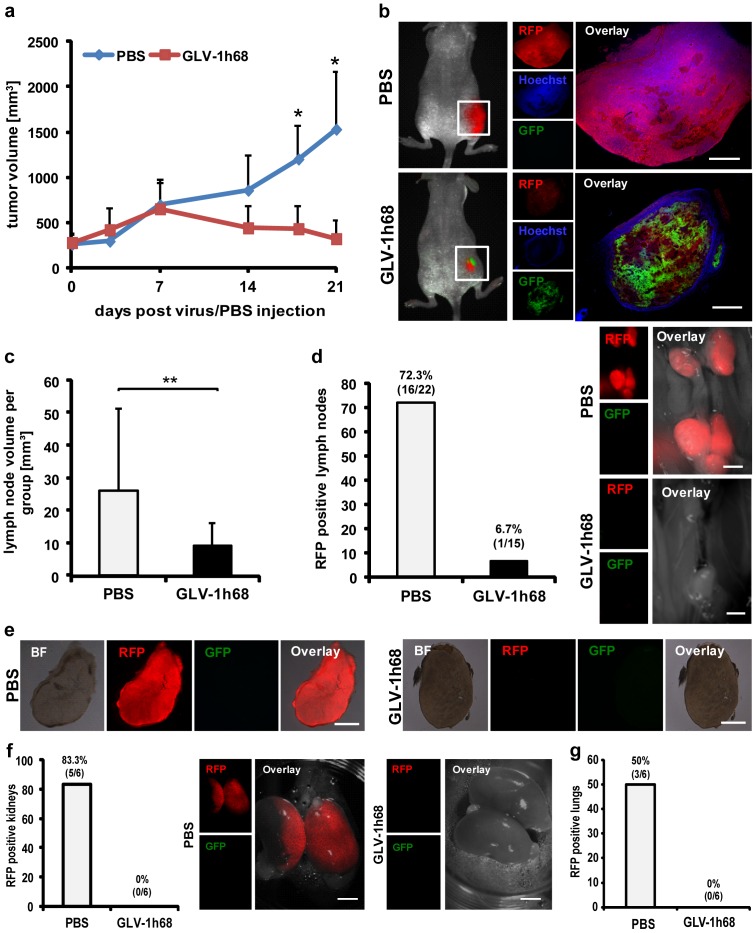
Influence of oncolytic virotherapy on C33A-RFP tumors and metastases. **a** Time curve of C33A-RFP tumor growth in 12 mice after administration of the oncolytic vaccinia virus GLV-1h68 and PBS, respectively (n = 6). Analyses of C33A-RFP tumors and metastases in **b-g** were done 32 dpti and 21 days after administration of GLV-1h68 or PBS (dpi). Six mice were examined per group (n = 6). **b** Left: C33A-RFP tumor-bearing mice 21 dpi. Right: 100 µm sections of a PBS and a GLV-1h68 treated tumor. Nuclei were stained with Hoechst dye, GFP is expressed by GLV-1h68 and RFP by C33A cells. **c** Volume of lumbar and renal lymph nodes and **d** percentage of RFP positive lymph nodes in PBS and GLV-1h68 treated C33A-RFP tumor-bearing mice (left). Right: Images of lumbar and renal lymph nodes in PBS and GLV-1h68 treated mice. **e** 100 µm sections of a PBS (left) and a GLV-1h68 (right) treated lumbar lymph node metastasis. **f** Left: Percentage of kidneys positive for RFP in PBS and GLV-1h68 treated mice. Right: Images of kidneys in the PBS and virus group. **g** Percentage of lungs positive for RFP in PBS and GLV-1h68 group. All images are representative examples. All scale bars represent 2 mm.

Once, metastatic spread of C33A-RFP cells in nude mice after s.c. implantation of tumor cells was characterized, we set out to analyze the possibility of an oncolytic virotherapy as a novel treatment option for C33A-RFP tumors and metastases. Recently, it has been shown, that the oncolytic vaccinia virus GLV-1h68 is an efficient agent in fighting hematogenous as well as lymphatic metastases in the human prostate cancer model PC-3 [22], [27]. Based on this, GLV-1h68 seems to be a promising candidate to eradicate C33A-RFP metastases. Initially, 12 C33A-RFP tumor-bearing mice were injected 11 dpti with 5×10^6^ plaque forming units (pfu) of GLV-1h68 or PBS as a control, respectively. As early as 14 days after virus/PBS injection (dpi) tumor volume in the GLV-1h68 treated group started to decrease. From 18 dpi on a statistical significant reduction of the C33A-RFP tumor volumes was achieved due to virus treatment when compared to the PBS treated control tumors (Fig. 6a). Tumor volumes were reduced to the initial size. Histological analyses of virus treated and control tumors 21 dpi revealed a massive spreading of GLV-1h68 in tumors of the virus group, associated with a lower fluorescence intensity of RFP and Hoechst, indicating virus-mediated, pronounced necrosis of tumor tissue (Fig. 6b). Furthermore, volumes of lumbar and renal lymph nodes in the PBS group were significantly higher when compared to those in the virus treated group 21 dpi, indicating a metastases inhibiting effect of GLV-1h68 (Fig. 6c). In further analyses, lymph nodes positive for C33A-RFP were determined by fluorescence microscopy. It turned out that 16 out of 22 enlarged lymph nodes (72.3%) were positive for RFP in the PBS group, whereas only 1 out of 15 enlarged lymph nodes (6.7%) was tested positive for RFP in the GLV-1h68 treated group (Fig. 6d), demonstrating the virus-mediated therapeutic effect on lymph node metastasis formation. Moreover, microscopical analyses of lumbar lymph node metastasis sections showed that neither RFP nor virus encoded GFP is detectable 21 days after virus injection. In contrast to this, a strong RFP signal was detected in lymph nodes of PBS treated C33A-RFP tumor bearing mice. Eventually, RFP in kidneys and lungs of C33A-RFP tumor bearing mice was analyzed microscopically and compared in PBS and virus treated groups. In both cases a drastic reduction of RFP was observed due to virus treatment (Fig. 6f, g).

Altogether, we demonstrated an efficient virus-mediated reduction of tumor size and metastatic burden in C33A-RFP tumor-bearing mice 21 dpi.

## Discussion

Since metastatic spread of carcinomas represents the major cause of cancer related death, there is an urgent need to intensify research focusing on the field of tumor metastasis. A lot of effort has been put into establishing metastatic models. Here, we describe the yet unpublished metastasizing ability of the HPV negative human cervical cancer cell line C33A in athymic nude mice after subcutaneous implantation of tumor cells, offering a new platform to analyze cancer metastasis and to test novel treatment modalities.

First of all, we observed an enlargement of lumbar and renal lymph nodes in C33A tumor-bearing mice (data not shown). Volume expansion of lymph nodes might be caused by an increasing amount of immigrating C33A cells, by an invasion of immune cells and/or might be a consequence of the premetastatic state of lymph nodes, which is due to hyperplasia and excessive proliferation of the lymph sinus endothelium. By inserting the cDNA encoding for *mRFP1* into the C33A cell genome, we generated a simple optical method to visualize metastatic spread of RFP positive tumor cells. In this study, we followed the fate of C33A-RFP cells after generation of subcutaneous tumors in athymic mice. In case of C33A-RFP tumor-bearing mice, RFP expressing tumor cells were detected in lumbar and renal lymph nodes, demonstrating that the enlargement of lymph nodes was mainly caused by metastasizing tumor cells. The constantly increasing amount of RFP positive lymph nodes over time showed a steady progression of lymph node metastases in correlation with tumor growth. Since we detected metastasized C33A-RFP cells 7 dpti only in LN1, this lymph node seemed to be the first target of metastasizing tumor cells, indicating metastasis of regional draining lymph nodes, a frequently described phenomenon for lymphatic metastasis [28]–[30]. The spread of cervical carcinomas to regional lymph nodes is a common hallmark of metastases and one of the primary determinants of outcome for patients [3], [4]. Moreover, at later stages we detected C33A-RFP cells in lymph vessels connecting regional lumbar and distant renal lymph node metastases. Thereby, we demonstrated that metastatic tumor cells can enter the lymphatic circulation and produce additional metastases, a process known as metastasis of metastases [31]. This pattern of metastatic tumor cell spread within the lymph system is a well described feature of many carcinomas [32]–[33].

Besides lymphatic metastases, we demonstrated the hematogenous spread of tumor cells in C33A-RFP tumor-bearing mice. Migrating C33A-RFP cells were on the one hand directly detected in blood vessels and on the other hand RFP positive micrometastases were observed in lungs of tumor-bearing mice. We further observed a very strong RFP signal in kidneys of C33A-RFP tumor-bearing mice. Histological analyses revealed an organized cellular localization of RFP in C33A cells in tumors, LNs and RNs, whereas RFP in kidneys was mainly located in the cortex area in spot-like patterns. A massive growth of primary tumors often leads to the development of hypoxic tumor regions. Hypoxia can influence tumor cells by acting as a stressor that impairs growth or causes cell death [34]. Dying C33A-RFP cells release red fluorescent proteins into the blood stream. As blood is filtered in the kidney nephrons, RFP can possibly be deposited in those structures. Furthermore, by FACS analyses and a blasticidin-survival study we assessed that the amount of viable C33A-RFP cells in kidneys was relatively small in comparison to the observed strong RFP signal. Therefore, we assume that the RFP signal in kidneys is caused in a higher degree by deposition of RFP than by the low number of RFP expressing metastasized tumor cells. Moreover, RFP might be associated with cell fragments, or taken up in vesicles.

In contrast to lungs and kidneys with only 5000 and 8000 viable tumor cells per gram tissue, respectively, about 15 million viable C33A-RFP cells per gram were present in lumbar and renal lymph nodes, suggesting lymph nodes as the main targets of metastasizing tumor cells in the C33A model. Lymphatic metastasis is known to be the main metastatic route of many cervical carcinomas in patients [17], [35].

Besides characterizing the metastatic behavior of C33A-RFP cells in nude mice, we analyzed the potential of oncolytic virotherapy for the treatment of primary tumors and their metastases in this model. While oncolytic virotherapy was already tested as a treatment option in C33A tumor-bearing mice, the therapeutic effect on metastases was not investigated. For example, Unno *et al*. treated cervical cancer with Sindbis virus [36] and Kim *et al*. analyzed the effect of an oncolytic adenovirus on cervix carcinomas [37]. Here, we set out for the first time to study the influence of the previously described oncolytic vaccinia virus GLV-1h68 [20]–[21] on C33A-RFP tumors as well as metastases. We observed a drastic reduction of the primary C33A-RFP tumor size due to virus treatment, indicating that this model is –responding well to vaccinia virus-mediated oncolytic virotherapy. Comparable treatment responses have been demonstrated in a variety of other tumor models, for example, in human malignant pleural mesothelioma [38], human pancreatic tumor xenografts [23] or anaplastic thyroid cancer [39]. A first indicator for an anti-metastatic effect of GLV-1h68 was evident by a significant decrease of the lymph node volume following virus treatment. Furthermore, a radical reduction of lymph nodes positive for metastasized C33A-RFP cells was observed in the virus-treated mice 21 dpi. Histological anaylsis revealed that neither RFP nor virus-encoded GFP signals were detectable in the majority of lymph nodes after virus treatment. We assume, on the one hand, that the massive viral colonization of the primary tumor caused a reduction of C33A-RFP cell migration to lymph nodes, and on the other hand that GLV-1h68 infection resulted in destruction of the already migrated and settled C33A-RFP cells in lymph nodes. Hence, there was an efficient diminishment of RFP expressing tumor cells in lumbar and renal lymph nodes by virus treatment. Without C33A-RFP tumor cells in the lymph nodes GLV-1h68 has no target structures to replicate in, explaining the absence of a GFP signal. In addition, a drastic virus-mediated reduction of RFP in lungs and kidneys of C33A-RFP tumor-bearing mice was demonstrated. As discussed above, the RFP reduction in kidneys might not be necessarily caused only by elimination of metastatic cells. In fact, we assume that there is a decrease in RFP deposition in kidneys after blood filtering, since the majority of RFP expressing tumor cells in mice seemed to be diminished earlier than three weeks after virus injection, due to viral infection and elimination of RFP positive cells. All in all, we showed that the oncolytic vaccinia virus GLV-1h68 has a great therapeutic potential in treating tumors as well as metastases of the cervical cancer cell line C33A.

## Conclusions

Since metastatic carcinomas represent a major health problem, it is crucial to intensify metastasis research. Here, we characterize the yet unknown metastatic behavior of the human cervical cancer cell line C33A in nude mice. We showed that C33A-RFP cells migrate from primary tumors at the flank of nude mice to lumbar and renal lymph node pairs, resulting in formation of lymph node metastases. The progression of lymph node metastases correlated with tumor growth, as seen in the clinic. Besides predominantly occurring lymphatic metastases, hematogenous spreading of C33A-RFP cells was detected. Furthermore, we analyzed the influence of oncolytic virotherapy on C33A-RFP tumors and metastases and demonstrated an efficient virus-mediated reduction of tumor size as well as metastatic burden.

Taken together, we hypothesize, that the C33A(-RFP) model might serve as a new platform to analyze cancer metastases and to test novel treatment modalities to efficiently fight metastases.

## Supporting Information

Table S1 HPV-status of different cervical cancer cell lines and their response to oncolytic vaccinia virus therapy. Different cervical cancer cell lines were screened for the effects of oncolytic virus therapy with GLV-1h68. The plus (+) in the second column indicates the presence of HPV DNA in cancer cells, minus (-) the absence. Plus in the last column means a successful regression of the tumor after virus administration, while minus in this column indicates that virus administration had no effects on tumor growth.
(DOCX)
