# Supplementary material for: Characterization of Metastasis Formation and Virotherapy in the Human C33A Cervical Cancer Model
Source: PLoS One. 2014 Jun 2;9(6):e98533. doi: 10.1371/journal.pone.0098533 (PMC4041767; doi:10.1371/journal.pone.0098533)
Supplement: Table S1 — HPV-status of different cervical cancer cell lines and their response to oncolytic vaccinia virus therapy. Different cervical cancer cell lines were screened for the effects of oncolytic virus therapy with GLV-1h68. The plus (+) in the second column indicates the presence of HPV DNA in cancer cells, minus (-) the absence. Plus in the last column means a successful regression of the tumor after virus administration, while minus in this column indicates that virus administration had no effects on tumor growth. (DOCX) [file pone.0098533.s001.docx]

**Supporting Information**

**Supplementary Material and Methods**

**Cell lines**

The HPV-positive human cervical cancer cell line SiHa was purchased from ATCC and cultured in EMEM (PAA Laboratories, Cölbe, Germany) supplemented with 10% FCS, 1% non-essential amino acids (PAA Laboratories, Cölbe, Germany) and 1% sodium pyruvate (Sigma-Aldrich, Steinheim, Germany). The HPV-positive human cervical cancer cell line CaSki was purchased from ATCC as well and cultured in RPMI-1640 (PAA Laboratories, Cölbe, Germany) with 10% FCS, 1% glucose (Sigma-Aldrich, Steinheim, Germany), 1% HEPES (PAA Laboratories, Cölbe, Germany), 1% sodium pyruvat and 1% L-glutamine (PAA Laboratories, Cölbe, Germany). The HPV-positive human cervical cancer cell line HeLa was obtained from ECACC and cultured in DMEM High Glucose supplemented with 10% FCS and 1% Penicillin/Streptomycin (PAA Laboratories, Cölbe, Germany).

**Ethics statement**

All animals were cared for and handled in strict accordance with good animal practices as defined by the national and local animal welfare bodies (Guide for the Care and Use of Laboratory Animals published by the National Institutes of Health and the German Animal Welfare Act “TierSchG”). Experimental protocols were approved by the government of Unterfranken, Germany (protocol numbers 55.2-2531.01-17/08, 55.2-2531.01-25/12 and 55.2-2531.01-38/12) and/or the Institutional Animal Care and Use Committee (IACUC) of Explora BioLabs, located in San Diego Science Center (San Diego, USA) (protocol numbers: EB08-003; EB11-025).

**Tumor implantation and virus administration**

Tumors were generated by implanting the cells in 100 µL PBS subcutaneously into the right abdominal flank of 6-8 weeks old female athymic nude *Foxn1^nu^* mice. 3 x 10^5^ (Caski), 5 x 10^6^ (SiHa) or 3 x 10^6^ (HeLa) cells were implanted per mice. When tumor volume reached 200-250 mm^3^ (CaSki after 20 days post implantation, SiHa after 34 days and HeLa after 19 days) mice were injected intravenously with 5 x 10^6^ pfu GLV-1h68 or 100 µl PBS (n=6). Tumor volume was measured over 48 dpi (CaSki), 56 dpi (SiHa) and 9 dpi (HeLa).

**Supplementary Table S1**

Table S1: **HPV-status of different cervical cancer cell lines and their response to oncolytic vaccinia virus therapy**

| **Cell line** | **HPV status** | **HPV DNA copy number** | **Tumor regression after GLV-1h68 administration** |
| --- | --- | --- | --- |
| CaSki | + | HPV-16, about 600 copies | + |
| SiHa | + | HPV 16, 1-2 copies | - |
| HeLa | + | HPV 18, about 30 copies | - |
| C33A | - | 0 | + |

Different cervical cancer cell lines were screened for the effects of oncolytic virus therapy with GLV‑1h68. The plus (+) in the second column indicates the presence of HPV DNA in cancer cells, minus (-) the absence. Plus in the last column means a successful regression of the tumor after virus administration, while minus in this column indicates that virus administration had no effects on tumor growth.

**Summary**

The results indicate that the presences and the copy number of HPV DNA in cervical cancer cells did not have an obvious impact on the therapeutic efficacy of GLV-1h68.
